# Using BD Vacutainer CD4 Stabilization Tubes for Absolute Cluster of Differentiation Type 4 Cell Count Measurement on BD FacsCount and Partec Cyflow Cytometers: A Method Comparison Study from Zimbabwe

**DOI:** 10.1371/journal.pone.0136537

**Published:** 2015-08-21

**Authors:** Florian Vogt, Rafael Van den Bergh, Andrea Bernasconi, Buhlebenkosi Moyo, Liberty Havazvidi, Mathieu Bastard, Laurence Flevaud, Fabian Taziwa, Eliphas Makondo, Sekesai Mtapuri-Zinyowera

**Affiliations:** 1 Operational Centre Barcelona, Médecins Sans Frontières / Doctors Without Borders, Barcelona, Spain; 2 Operational Research Unit Luxembourg, Médecins Sans Frontières / Doctors Without Borders, Luxembourg, Luxembourg; 3 Department of Field Epidemiology and Training, Epicentre, Paris, France; 4 Beitbridge Project, Médecins Sans Frontières / Doctors Without Borders, Beitbridge, Zimbabwe; 5 Zimbabwe Mission, Médecins Sans Frontières / Doctors Without Borders, Harare, Zimbabwe; 6 Department of Clinical Research, Epicentre, Paris, France; 7 Laboratory Department, Beitbridge District Hospital, Ministry of Health and Child Welfare, Beitbridge, Zimbabwe; 8 National Microbiology Reference Laboratory, Ministry of Health and Child Welfare, Harare, Zimbabwe; Ghent University, BELGIUM

## Abstract

**Background:**

Blood collected in conventional EDTA tubes requires laboratory analysis within 48 hours to provide valid CD4 cell count results. This restricts access to HIV care for patients from rural areas in resource-constraint settings due to sample transportation problems. Stabilization Tubes with extended storage duration have been developed but not yet evaluated comprehensively.

**Objective:**

To investigate stability of absolute CD4 cell count measurement of samples in BD Vacutainer CD4 Stabilization Tubes over the course of 30 days.

**Methods:**

This was a laboratory-based method comparison study conducted at a rural district hospital in Beitbridge, Zimbabwe. Whole peripheral blood from 88 HIV positive adults was drawn into BD Vacutainer CD4 Stabilization Tubes and re-tested 1, 2, 3, 5, 7, 14 and 30 days after collection on BD FacsCount and Partec Cyflow cytometers in parallel. Absolute CD4 cell levels were compared to results from paired samples in EDTA tubes analysed on BD FacsCount at the day of sample collection (references methodology). Bland-Altman analysis based on ratios of the median CD4 counts was used, with acceptable variation ranges for Limits of Agreements of +/-20%.

**Results:**

Differences in ratios of the medians remained below 10% until day 21 on BD FacsCount and until day 5 on Partec Cyflow. Variations of Limits of Agreement were beyond 20% after day 1 on both cytometers. Specimen quality decreased steadily after day 5, with only 68% and 40% of samples yielding results on BD FacsCount and Partec Cyflow at day 21, respectively.

**Conclusions:**

We do not recommend the use of BD Vacutainer CD4 Stabilization Tubes for absolute CD4 cell count measurement on BD FacsCount or Partec Cyflow due to large variation of results and decay of specimen quality. Alternative technologies for enhanced CD4 testing in settings with limited laboratory and sample transportation capacity still need to be developed.

## Introduction

For HIV infected persons, in particular in resource-limited settings, treatment decisions are largely contingent on absolute CD4+ T-lymphocyte cell counts in venous blood. This measurement serves as eligibility criteria for anti-retroviral therapy (ART), for monitoring treatment response in settings where viral load testing is not routinely available as recommended by the World Health Organisation, and is used to determine eligibility for prophylactic treatment of opportunistic infections by *Pneumocystis carinii*, *Toxoplasma gondii*, and *Cryptococcus neoformans* [1–3]. Zimbabwe, as most Sub-Saharan African (SSA) countries, gives priority for ART initiation to patients below 350 cells/μL, and CD4 testing rather than viral load quantification continues to be the method of choice for clinical decision making [4]. Thus, for many HIV infected people worldwide, access to CD4 testing remains a prerequisite for access to treatment.

CD4 testing capacity, however, has not kept the same pace as ART roll-out in many developing countries, including Zimbabwe [5]. High costs of laboratory equipment, lack of skilled staff, and poor health centre infrastructure often restrict CD4 diagnostics to centralized laboratories in cities, resulting in the need to transport samples from rural to urban facilities [6–9]. Transport is difficult and costly in many resource-limited settings. Blood collected in Ethylenediaminetetraacetic acid (EDTA) tubes, of widespread use for whole blood sample collection across SSA countries, needs to be analysed within 48 hours to guarantee valid results [10]. This is not always feasible in many developing countries, especially in remote rural areas, which negatively affects CD4 testing coverage and ultimately treatment initiation and retention in care [11, 12].

Significant advances in CD4 testing technology have been made over the past two decades [13]. Today, the most commonly used method is flow cytometry [14–16]. State-of-the-art dedicated single-platform flow cytometers are highly automated, robust, have high throughput capacity and provide direct absolute CD4 counts whilst being easy to operate, which makes them suitable for high-burden, resource-limited settings [17]. Among these models, the BD FacsCount (Becton Dickinson, Franklin Lakes, New Jersey, USA) is considered as reference methodology, and is together with the Partec Cyflow (Partec GmbH, Muenster, Germany) the most commonly used flow cytometer in SSA countries today [18, 19].

In 2006, new sample collection tubes (BD Vacutainer CD4 Stabilization Tubes (STs), Becton Dickinson, Franklin Lakes, New Jersey) were developed. STs contain a cell preservative in addition to EDTA to conserve qualitative and quantitative leukocyte characteristics. According to the manufacturer, STs can preserve whole venous blood for up to 3 days at 37°C or up to 7 days at 30°C [20, 21]. This bears promising potential to circumvent the need for fast sample transportation.

To our knowledge, only one study has been published evaluating STs for CD4 measurement, using a BD FacsCount flow cytometer [22]. While results of this study were reported as promising, the sample size of 59 specimens was relatively small, the study duration was limited to 8 days, and the statistical analysis was not comprehensive. Evidence for performance of STs in combination with other common flow cytometers such as the Partec Cyflow is entirely non-existent.

We therefore conducted a prospective laboratory evaluation study to investigate for how long blood samples collected in STs can be used for CD4 testing. ST samples of HIV positive patients were re-tested over 30 days on BD FacsCount and Partec Cyflow cytometers in parallel, and absolute CD4 cell counts were compared against standard EDTA tube results from the day of sample collection (reference methodology).

## Material and Methods

### Setting

This study was carried out in Beitbridge district, Zimbabwe, where Médecins Sans Frontières (MSF) supported the Ministry of Health and Child Welfare in the prevention, treatment and care of HIV/AIDS at Beitbridge District Hospital (BDH) Opportunistic Infections (OI) clinic and six rural health centres during 2009–2013. Beitbridge district is situated in Southern Zimbabwe forming borders with South Africa. It has a population of about 122,000 and an HIV prevalence of 21.2% among the adult population, which is substantially higher than the national average of 15.2% [23, 24]. The BDH laboratory is the only public facility in the district providing comprehensive laboratory diagnostics for HIV. It is equipped with two cytometers, a BD FacsCount (Ref-No 337858) and a Partec Cyflow SL_3 (Ref-No CY-S-1023) cytometer. Both instruments are used concurrently for CD4 testing. Standard EDTA tubes are almost universally used for CD4 sample collection in Beitbridge. STs have been available in Zimbabwe since 2012 but are not widely used.

### Study population

All HIV positive patients aged ≥18 years that came to BDH OI clinic with routinely scheduled CD4 testing appointments during the study period for either ART eligibility assessment or routine treatment monitoring were eligible. We aimed at recruiting 90 participants.

### Study conduct

#### Recruitment and sample collection

Recruitment occurred in consecutive order of appointment at all week days during the study period upon opening of BDH OI clinic in the morning. All eligible patients were invited and were asked to provide written informed consent. The target number for daily enrolment was decided each day according to the work load in order not to interfere with standard patient care. Two whole venous peripheral blood samples, 4 ml in EDTA tubes as per routine practice and 2 ml in STs for study purposes, were drawn using standard phlebotomy techniques. All tubes were inverted 10 times immediately after drawing, placed in vertical racks and brought to the BDH laboratory. Patients´ age, sex and ART treatment status was also recorded at enrolment.

#### Storage and re-testing of samples

At the day of sample collection (day 0), both tubes from each patient were analysed on BD FacsCount and Partec Cyflow within 6 hours after bleeding. EDTA tube samples were not re-tested and were disposed the same day. ST samples were kept and aliquots were tested on both cytometers in parallel at 1, 2, 3, 5, 7, 14, 21 and 30 days after bleeding. Samples were tested only once on each cytometer at each of these days. STs were stored in vertical racks inside the BDH laboratory between 25–30°C in the dark with free circulation of ambient air and were inverted at least 10 times immediately each time before being re-tested. All manufacturer instructions were followed for the operation of instruments and use of devices during this study.

#### CD4 testing on BD FacsCount

The BD FacsCount is a fully automated micro-bead based benchtop cytometer equipped with a green laser for detection of PE and PE.Cy5 labels after no-wash no-lyse sample preparation. We used single-tube BD FacsCount reagents (Ref-No 339010) containing reference beads and monoclonal antibodies conjugated with CD4 PE, CD14 PE-Cy5, and CD15 PE-Cy5 fluorochromes. In short, 50 μL of whole blood was added to the reagent tube using reverse pipetting technique. After 30 minutes incubation in the dark, 50 μL of 5% formaldehyde fixative solution was added before analysis. Gating was done automatically by the built-in BD FacsCount software (Ref-No 339011).

#### CD4 testing on Partec Cyflow

Partec Cyflow is based on true volumetric absolute counting without the need for reference beads [14, 17]. The Partec Cyflow SL_3 used in this study detects three parameters (side scatter and two fluorochromes) following a no-wash no-lyse protocol. Through reverse pipetting, 20 μL of Partec Easy Count reagent kit (Ref-No 05_8401) containing monoclonal antibodies with PE fluorochromes was added to 20 μL of patient blood in Partec sample tubes (Ref-No 04–2000). After 15 minutes incubation in the dark, 800 μL no-lyse buffer was added prior to analysis. Data analysis was done using Windows-based Partec FloMax software v.1.4. Gating was done manually on the FL2-SSC scatter plot.

#### Recording of results

Clotting resulted in exclusion from further re-testing due to potential damage to the cytometers. Samples with visible haemolysis were not tested. No results were recorded for samples rejected automatically by BD FacsCount for quality reasons. For Partec Cyflow, the quality of cell separation was classified as good, acceptable or poor. Only results with good or acceptable cell separation were kept. CD4 test results were recorded into a paper-based logbook directly after testing and concurrently entered into a MS Excel-based study database.

#### Study quality provisions

Detailed standard operating procedures were developed and all study staff were trained on the study documents. A pilot was done for improved conduct of the actual study. BDH OI guidelines and BDH laboratory standards were followed for phlebotomy and laboratory procedures. Sample testing was done by one experienced laboratory technician following the manufacturers´ operating guidelines. The same lots of reagents and other consumables were used during the study period. Internal quality controls were run daily on both cytometers and Levey–Jennings charts were drawn [25, 26]. The BDH laboratory participates in the external quality control scheme of the Zimbabwe National Quality Assurance Programme. At the end of the study conduct, each data point in the study database was verified by two persons independently against the original data sources.

### Analysis

ST performance was analysed for BD FacsCount and Partec Cyflow separately by comparing ST results at each re-testing day (1, 2, 3, 5, 7, 14, 21 and 30 days after sample collection) with paired BD FacsCount EDTA sample results from day 0 (reference test) for each cytometer. Additionally, differences among cytometers were assessed over time by comparing ST results from the same re-testing day between cytometers. Medians and Inter-Quartile-Ranges (IQRs) were used as descriptive summary statistics.

Bland-Altman agreement assessment was chosen as the primary method of analysis [27]. Logarithmic transformation was done for not normally distributed differences between paired samples or if differences varied along CD4 levels, and the ratio of the geometric means including 95% Limits of Agreements (LoAs) was calculated after back-transformation [28–33]. Since the geometric mean is an approximation of the median, agreement was expressed as ratios of the medians [34]. Crude analysis irrespective of CD4 levels was done, as well as by subgroup with ≤350 and ≤500 cells/μL cut-offs as currently recommended for clinical decision making [1, 4]. Data were censored if the number of observations was ≤10% of the original sample size [35].

For separate cytometer analyses, agreement ranges were decided *a priori* as “good” if LoAs ranged within 10% above or below the EDTA sample result on BD FacsCount at day 0 (reference test), as “acceptable” for ranges between 10–20%, and as “unacceptable” if LoAs exceeded 20% above or below that reference value [36–38]. We used the acceptable variability of +/-20% as threshold to determine whether or not STs were considered suitable for sample storage. This choice was guided by clinical judgement, the relatively low inherent variability of 5% for BD FacsCount and 2% for Partec Cyflow reported by the manufacturers [39, 40], and by acceptability thresholds of 10% and 15% used to assess stability of EDTA tubes on BD FacsCount over 24 and 48 hours, respectively [39]. For cytometer comparison over time, LoAs were compared against agreement ranges from the same re-testing day.

As secondary methods of analysis, the percentage of correctly classified patients using STs was calculated for ART eligibility thresholds 350 and 500 cells/μL compared to EDTA sample results on FacsCount at day 0. Additionally, Pearson correlation coefficients and mean percentage similarities including coefficients of variation were calculated by comparing results from each cytometer at each re-testing day separately against the reference test [41–43]. Furthermore, a random effects linear model was fitted to assess cytometer effects on CD4 trends and differences according to the cytometer used [44].

### Ethics

Ethics approval was obtained from the Medical Research Council of Zimbabwe (Harare, Zimbabwe) and from the Médecins Sans Frontières Ethics Review Board (Geneva, Switzerland). Written informed consent was obtained from each participant prior to enrolment. All patients at the BDH OI clinic received regular standard of care during the study period irrespective of participation in this study.

## Results

Recruitment and sample collection took place between 9 and 24 October 2013, with a minimum of four and a maximum of 19 enrolments per day. Overall, 91 patients attending BDH OI clinic were screened and found eligible. Of these, two patients refused to participate and the informed consent form of another participant was lost during the enrolment process, leaving samples from 88 patients for analysis. Among these, 64 (73%) were female and 24 (27%) were male, and 52 (59%) participants were on ART while 36 (41%) were not on ART. The median age among study participants was 35.5 years (IQR 31.0–42.5) (Table 1). The median CD4 count of BD FacsCount EDTA samples at day 0 (reference test) was 459 cells/μL (IQR 281–590) with a range from 12 to 1,194 cells/μL.

**Table 1:** Characteristics of study participants at enrolment.

	N	(%) ^a^
Sex		
female	64	(72.7)
male	24	(27.3)
Age *(years)*		
<30	13	(14.8)
30–39	42	(47.7)
40–49	23	(26.1)
>50	10	(11.4)
Median (IQR)	35.5 (31–42.5)	
Treatment status		
on ART	52	(59.1)
not on ART	36	(40.9)

^a^ Percentage of column totals.

ART: Anti-Retroviral Therapy; IQR: Inter-Quartile Range; N: Number of patients.

**Fig 1:**
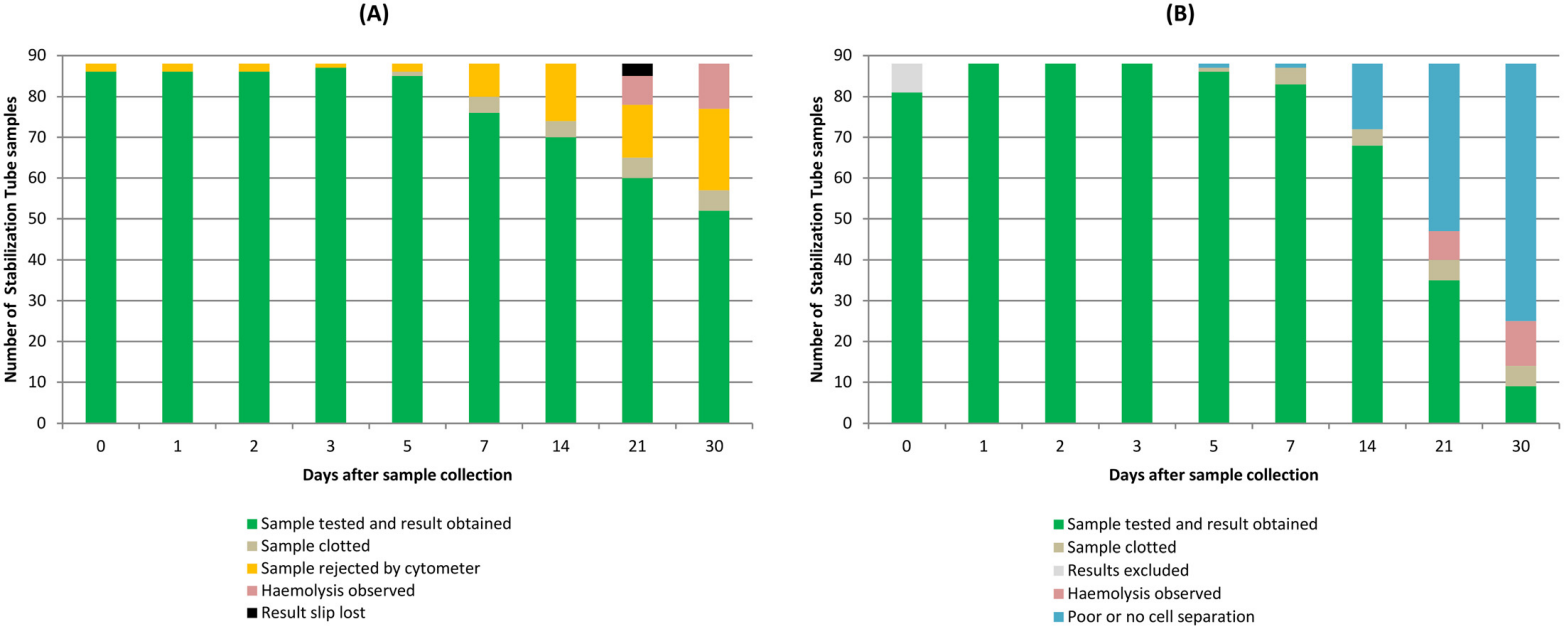
BD Vacutainer CD4 Stabilization Tube samples retained and results obtained over time. (A) Number of BD Vacutainer CD4 Stabilization Tube samples on BD FacsCount at days 0–30. (B) Number of BD Vacutainer CD4 Stabilization Tube samples on Partec Cyflow at days 0–30.

Five samples had to be excluded due to clotting during the re-testing period. Among the 83 samples retained until day 21, results could be obtained for 60 (68.1%) samples on BD FacsCount and the 35 (39.7%) samples on Partec Cyflow. At day 30, analysis on BD FacsCount yielded 52 results, and nine results on Partec Cyflow. The latter were censored due to small sample size. Main reasons for not obtaining results were haemolysis, sample rejection on BD FacsCount, and poor cell separation quality on Partec Cyflow (Fig 1).

### CD4 cell counts from STs samples on BD FacsCount

**Fig 2:**
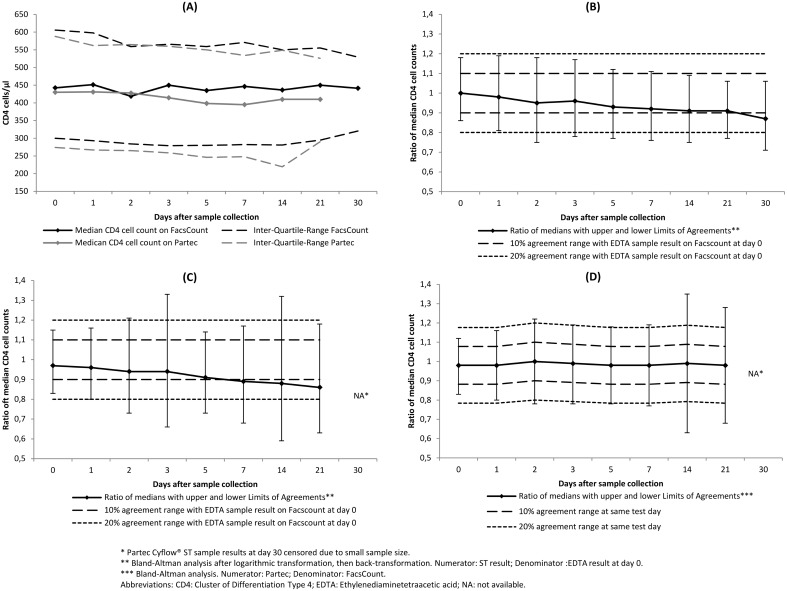
Stability of BD Vacutainer CD4 Stabilization Tube sample results over time. (A) Absolute CD4 cell counts of BD Vacutainer CD4 Stabilization Tube samples on BD FacsCount and Partec Cyflow at days 0–30. (B) Agreement of BD Vacutainer CD4 Stabilization Tube sample results on BD FacsCount at days 0–30 with EDTA sample results on BD FacsCount at day 0. (C) Agreement of BD Vacutainer CD4 Stabilization Tube sample results on Partec Cyflow at days 0–30 with EDTA sample results on BD FacsCount at day 0. (D) Agreement of BD Vacutainer CD4 Stabilization Tube sample results on Partec Cyflow at days 0–30 with same day results of BD Vacutainer CD4 Stabilization Tube sample results on BD FacsCount.

The median CD4 count for BD FacsCount ST samples was stable throughout the re-testing period, with 442.5 cells/μL (IQR 300–606) at day 0 and 441.5 cells/μL (IQR 320.5–529.5) at day 30. The lowest median was recorded at day 2 (419 cells/μL (IQR 284–559) (Fig 2 part A).

Bland-Altman analysis was done with log-transformed values because of not-normally distributed differences. The ratios of the median ST and the median EDTA results at day 0 decreased steadily from 1.0 (LOAs 0.86–1.18) at day 0 to 0.87 (LoAs 0.71–1.06) at day 30, but was above 0.9 until day 21. At none of the re-testing days were both LoAs within the 10% range. At day 0 and 1, both the upper and the lower LoAs were within the 10–20% range. Beyond day 1, the lower LoA was constantly outside the 20% range, whereas the upper LoA remained inside (Fig 2 part B). Subgroup analysis with cut-offs at 350 and 500 cells/μL showed similar patterns.

The percentage of correctly classified patients using STs remained above 90% on most days for ART eligibility threshold ≤350 CD4 cells/μL with the lower limits of the confidence intervals reaching as low as 69.9% at day 21. A similar pattern was observed for ART eligibility threshold ≤500 CD4 cells/μL, where the lower limits of the confidence intervals reached as low as 59.0% at day 30 (Table 2). The Pearson correlation coefficient remained at 0.97 or higher until day 21 and then decreased to 0.95 at day 30 (Table 3). Mean percentage similarity decreased continuously from 100.43% (CV 4.05) at day 0 to 93.54% (CV 4.70) at day 30 (Table 4).

**Table 2:** Percentage of correctly classified BD Vacutainer CD4 Stabilization Tube sample results on BD FacsCount for ART eligibility thresholds of 350 and 500 CD4 cells/μL over time with EDTA tube results on FacsCount at day 0 as reference methodology.

	ART eligibility ≤ 350 CD4 cells/μL		ART eligibility ≤ 500 CD4 cells/μL	
Day	%	(CI)	%	(CI)
0	91.4	(77.6–97.0)	100.0	(90.1–100.0)
1	91.2	(77.1–97.0)	94.1	(80.9–98.4)
2	88.2	(73.4–95.3)	88.2	(73.4–95.3)
3	91.4	(77.6–97.0)	97.1	(85.5–99.5)
5	97.1	(85.1–99.5)	94.1	(80.9–98.4)
7	96.7	(83.3–99.4)	100.0	(88.6–100.0)
14	91.7	(74.2–97.7)	87.5	(69.0–95.7)
21	90.0	(69.9–97.2)	100.0	(83.9–100.0)
30	100.0	(81.6–100.0)	82.4	(59.0–93.8)

ART: Anti-retroviral therapy. CD4: Cluster of Differentiation Type 4. CI: Confidence Interval.

**Table 3:** Correlation of BD Vacutainer CD4 Stabilization Tube sample results on BD FacsCount and Partec Cyflow over time.

	BD FacsCount ^a^	Partec Cyflow ^a^	Partec Cyflow vs. BD FacsCount ^b^
Day	Correlation Coefficient ^c^	Correlation Coefficient ^c^	Correlation Coefficient ^c^
0	0.98	0.98	0.98
1	0.97	0.98	0.98
2	0.97	0.99	0.98
3	0.98	0.99	0.98
5	0.98	0.97	0.98
7	0.97	0.97	0.97
14	0.97	0.93	0.94
21	0.97	0.88	0.91
30	0.95	– ^d^	– ^d^

^a^ Compared with EDTA sample results on BD FacsCount at Day 0 (reference methodology).

^b^ Comparing same day results of BD Vacutainer CD4 Stabilization Tubes on BD FacsCount and Partec Cyflow cytometers.

^c^ Pearson product-moment correlation coefficient.

^d^ Partec Cyflow Stabilization Tube sample results at day 30 censored due to small sample size.

BD: Becton Dickinson; CD4: Cluster of Differentiation Type 4.

**Table 4:** Similarity of BD Vacutainer CD4 Stabilization Tube sample results on BD FacsCount and Partec Cyflow over time.

	BD FacsCount ^a^		Partec Cyflow ^a^		Partec Cyflow vs. BD FacsCount ^b^	
Day	MPSV	(CV)	MPSV	(CV)	MPSV	(CV)
0	100.43	(4.05)	98.92	(4.14)	98.52	(3.60)
1	99.42	(4.70)	98.35	(4.75)	98.85	(4.58)
2	97.59	(5.20)	97.49	(5.45)	100.98	(5.54)
3	98.44	(5.20)	97.46	(5.88)	99.65	(5.02)
5	96.76	(4.71)	95.80	(5.04)	99.02	(4.93)
7	96.25	(4.55)	94.84	(5.74)	99.03	(5.35)
14	95.47	(4.05)	95.09	(10.25)	99.69	(9.02)
21	95.62	(3.80)	93.76	(6.68)	98.30	(7.61)
30	93.54	(4.70)	– ^c^	– ^c^	– ^c^	– ^c^

^a^ Compared with EDTA sample results on BD FacsCount at Day 0 (reference methodology).

^b^ Comparing same day results of BD Vacutainer CD4 Stabilization Tubes on BD FacsCount and Partec Cyflow cytometers.

^c^ Partec Cyflow Stabilization Tube sample results at day 30 censored due to small sample size.

BD: Becton Dickinson; CD4: Cluster of Differentiation Type 4; CV: Coefficient of Variation; MPSV: Mean Percentage Similarity Value.

### CD4 cell counts from ST samples on Partec Cyflow

Seven Partec Cyflow ST samples at day 0 showed saliently low results compared to corresponding BD FacsCount EDTA tube and ST results (range of differences -601 to -223 cells/μL and -572 to -211 cells/μL, respectively). Further review of the data revealed that all these results belonged to a batch of samples that were tested in consecutive order at the same day. The most likely explanation for this anomalous discrepancy is a confusion of samples or a mistake in result recording at that day. Results were excluded from analysis.

The median CD4 count of Partec Cyflow ST samples was 430 cells/μL (IQR 274–588) at day 0 and decreased slightly to 410 cells/μL (IQR 290–526) at day 21 (Fig 2 part A).

As with BD FacsCount, log-transformation was indicated for Bland-Altman analysis of ST results on Partec Cyflow. Ratios of the median Partec Cyflow ST results and the median day 0 BD FacsCount EDTA tube results were 0.97 (IQR 0.83–1.15) at day 0 and decreased to 0.92 (IQR 0.72–1.18) at day 30. The lowest ratio was recorded at day 21 (0.86; IQR 0.63–1.18). The ratio of the median remained above 0.9 until day 5. At no re-testing day were either the upper or the lower LoAs within the 10% range. At day 0 and 1, both the upper and the lower LoAs were within the 10–20% agreement range. Beyond day 1, all lower LoAs were outside the 20% range while most upper LoAs remained inside (Fig 2 part C). Subgroup analysis for CD4 thresholds of 350 and 500 cells/μL showed similar patterns.

The percentage of correctly classified patients using STs remained above 90% on most days for ART eligibility threshold ≤350 CD4 cells/μL with the lower limits of the confidence intervals reaching as low as 56.6% at day 30. A similar pattern was observed for ART eligibility threshold ≤500 CD4 cells/μL, where the lower limits of the confidence intervals reached as low as 69.0% at day 14 (Table 5). The Pearson correlation coefficient remained stable and above 0.97 at all days except day 14 (0.93) (Table 3). Mean percentage similarity decreased from 98.92% (CV 4.14) at day 0 to 93.76% (CV 6.68) at day 21 (Table 4).

**Table 5:** Percentage of correctly classified BD Vacutainer CD4 Stabilization Tube sample results on Partec Cyflow for ART eligibility thresholds of 350 and 500 CD4 cells/μL over time with EDTA tube results on FacsCount at day 0 as reference methodology.

	ART eligibility ≤ 350 CD4 cells/μL		ART eligibility ≤ 500 CD4 cells/μL	
Day	%	(CI)	%	(CI)
0	96.7	(83.3–99.4)	100.0	(88.6–100.0)
1	94.3	(81.4–98.4)	100.0	(90.1–100.0)
2	97.1	(85.5–99.5)	97.1	(85.5–99.5)
3	88.6	(74.0–95.5)	100.0	(90.1–100.0)
5	97.1	(85.5–99.5)	91.4	(77.6–97.0)
7	100.0	(89.6–100.0)	90.9	(76.4–96.9)
14	91.7	(74.2–97.7)	87.5	(69.0–95.7)
21	100.0	(77.2–100.0)	100.0	(77.2–100.0)
30	100.0	(56.6–100.0)	- ^a^	- ^a^

^a^ No data available.

ART: Anti-retroviral therapy. CD4: Cluster of Differentiation Type 4. CI: Confidence Interval.

### Comparison of CD4 cell counts from STs samples on Partec Cyflow with BD FacsCount

The differences of the medians between Partec Cyflow ST samples and BD FacsCount ST samples at the same re-testing day varied between 9 cells/μL at day 2 and 51.3 cells/μL at day 7, with Partec Cyflow underreporting cell counts on most days (Fig 2 part A).

Bland-Altman analysis results of Partec Cyflow STs against BD FacsCount STs at the same re-testing day showed stable ratios of the medians between 0.98 (LoA 0.83–1.12) at day 0 and 0.98 (LOA 0.68–1.28) at day 21. At no re-testing day were either the upper or the lower LoAs within the 10% range of the same test day. Both LoAs were within the 10–20% range at day 0, 1 and 5, and outside the 20% range at all other days (Fig 2 part D).

The Pearson correlation coefficient remained above 0.97 until day 7 and then decreased to 0.91 at day 21 (Table 3). Mean percentage similarity remained high and stable above 98% throughout (Table 4).

Based on a random effects mixed model with random intercept and random slope, ST results were on average 8.32 cells/μL lower on Partec Cyflow than on BD FacsCount (95%CI 4.57–12.08; p-value <0.001), with an average reduction per day of 1.95 cells/μL (95%CI 1.51–2.40; p-value <0.001). No interaction effect between days and cytometer was found, suggesting no substantial effect of time on the difference between cytometers.

## Discussion

We conducted the most comprehensive method comparison study using STs for absolute CD4 cell testing to date, with findings applicable for two of the most common CD4 testing instruments in high HIV prevalence countries. After day 1, observed variations exceeded the pre-defined acceptable range of +/-20% on both cytometers, and results could thus not be considered to be in agreement with the gold standard beyond that point even though the point estimate of the agreement bias remained relatively small throughout the 30 day study period (Fig 2). We also noticed a decay in specimen quality beyond day 7 that resulted in an increasing number of samples for which no results could be obtained (Fig 1).

Our findings contradict statements by the manufacturer about STs [20] and also conclusions drawn in the manufacturer´s white paper on STs [21]. In that proof of principal study, “similar” CD4 count results for EDTA tubes and STs were reported for up to 5 days on a BD FacsCount cytometer for three different sample storage temperature ranges (2–8°C, 22–26°C, and 28–32°C). However, that study relied on blood samples from only three participants, all of which were healthy volunteers from BD Biosciences [21], and was not subject to a peer-review process.

Our results also contradict findings from the only other published STs evaluation study, which reported STs to “strongly agree” with EDTA tubes using BD FacsCount [22]. However, no Bland-Altman analysis and no pre-defined cut-offs were presented in that research to support this conclusion.

Misclassification analysis for the two most widely used ART eligibility thresholds in SSA of 350 and 500 CD4 cells/μL showed relatively high estimated proportions of patients correctly classified by STs as ART eligible. However, this study was not powered for this kind of analysis, which is shown by the wide confidence interval for all estimates. Results should hence be interpreted with appropriate caution.

A major strength of our study is that it was conducted under real operational conditions embedded into routine health services at a district level facility in a typical resource-limited high HIV burden setting. This, however, made repeated testing of samples on the same cytometer at the same day impossible due to high work load. Consequently, inter- or intra-run variability could not be assessed.

The study conduct coincided with the beginning of the summer rainy season in Zimbabwe. Temperature conditions up to 30°C as recorded during the study period are common in many SSA countries. The study duration was deliberately kept at a minimum to avoid confounding by changing environmental factors. The maximum temperature variation of 5°C during the study period confirmed this.

Our study population included HIV positive patients on ART and not on ART that came to BDH OI clinic with a routine appointment. While this might have led to an underrepresentation of rural or clinically unstable patients, there is no reason to assume differences in blood sample stability from these patients. Also, only two (2.2%) among all eligible patients refused to participate, making effects of selection bias further unlikely. Inclusion criteria were kept deliberately wide to allow generalizability of results to a broad target population.

Our study provides novel insights into why ST samples had to be excluded from testing and why results from retained ST samples could not be obtained. Only five samples clotted during the 30 days. However, sample quality decreased substantially over time, as could be seen by rising frequency of haemolysis, sample rejection by BD FacsCount, and increasingly poor quality of CD4+ T-lymphocyte cell separation on Partec Cyflow (Fig 1). The latter was the main reason to have day 30 ST results on Partec Cyflow excluded due to small sample size. This finding calls into question the utility of STs for storing whole blood sample, in particular for more than 5 days, independently from results obtained from retained samples.

The role of absolute CD4 cell count measurement in clinical HIV management is changing [45]. ART eligibility criteria are constantly being broadened while viral load technology is still out of financial reach for many developing countries [1, 43]. These factors might lead to a temporary increased demand for CD4 testing until prices for viral load testing have reduced sufficiently to replace CD4 testing for routine treatment monitoring in SSA countries. Also, there are a few promising point-of-care technologies under development [19, 46, 47], though evaluations from field settings show mixed results [48, 49]. This technology has the potential to make timely sample transportation between rural and central facilities obsolete. In the long run, CD4 testing is likely to become less frequent as a blanket diagnostic tool, but will remain important as eligibility assessment for ART and for preventive treatment of opportunistic infections [2, 3, 45]. STs could be an appropriate way to meet this temporary rise in CD4 testing rather than to continue installing costly benchtop flow cytometers in peripheral health centres that might lead to an excess CD4 test capacity in a few years.

For this, however, STs need to prove acceptable agreement with EDTA tubes on BD FacsCount at day of sample collection, the current reference methodology, for at least the period stated by the manufacturer [20, 21]. This was not the case in our study due to large variation of results. We therefore do not recommend the use of BD Vacutainer CD4 Stabilization Tubes for absolute CD4 cell count measurement on BD FacsCount or Partec Cyflow cytometers without conducting further evaluation research. Alternatives to improve access to CD4 testing for patients from rural areas in developing countries, where both sample transportation capacity as well as laboratory infrastructure is poor, are yet to be found.

## Supporting Information

S1 Table Agreement of BD Vacutainer CD4 Stabilization Tube sample results on BD FacsCount over time above and below 350 and 500 cells/μL cut-off levels with EDTA sample results on BD FacsCount at Day 0. (DOC)

S2 Table Agreement of BD Vacutainer CD4 Stabilization Tube sample results on Partec Cyflow over time above and below 350 and 500 cells/μL cut-off levels with EDTA sample results on BD FacsCount at Day 0. (DOC)

S3 Table BD Vacutainer CD4 Stabilization Tubes study database. (CSV)
