# Supplementary material for: Using BD Vacutainer CD4 Stabilization Tubes for Absolute Cluster of Differentiation Type 4 Cell Count Measurement on BD FacsCount and Partec Cyflow Cytometers: A Method Comparison Study from Zimbabwe
Source: PLoS One. 2015 Aug 21;10(8):e0136537. doi: 10.1371/journal.pone.0136537 (PMC4546686; doi:10.1371/journal.pone.0136537)
Supplement: S1 Table — (DOC) [file pone.0136537.s001.doc]

**S1_Table: Agreement of BD Vacutainer® CD4 Stabilization Tube sample results on BD FacsCount® over time above and below 350 and 500 cells/μL cut-off levels with EDTA sample results on BD FacsCount® at Day 0.**

|  | **CD4 < 350 cells/μL** | | **CD4 > 350 cells/μL** | | **CD4 < 500 cells/μL** | | **CD4 > 500 cells/μL** | |
| --- | --- | --- | --- | --- | --- | --- | --- | --- |
| **Day** | **Ratio of medians** ^a^ | **(LoA)** | **Ratio of medians** ^a^ | **(LoA)** | **Ratio of medians** ^a^ | **(LoA)** | **Ratio of medians** ^a^ | **(LoA)** |
| 0 | 1.03 | (0.88 – 1.21) | 0.99 | (0.85 – 1.15) | 1.01 | (0.86 – 1.19) | 0.99 | (0.85 – 1.17) |
| 1 | 0.99 | (0.84 – 1.17) | 0.98 | (0.80 – 1.21) | 0.99 | (0.83 – 1.17) | 0.97 | (0.79 – 1.21) |
| 2 | 0.98 | (0.83 – 1.15) | 0.93 | (0.72 – 1.19) | 0.96 | (0.76 – 1.21) | 0.92 | (0.75 – 1.15) |
| 3 | 0.99 | (0.78 – 1.26) | 0.94 | (0.80 – 1.12) | 0.98 | (0.79 – 1.23) | 0.94 | (0.81 – 1.09) |
| 5 | 0.94 | (0.77 – 1.14) | 0.93 | (0.76 – 1.12) | 0.94 | (0.78 – 1.14) | 0.92 | (0.76 – 1.11) |
| 7 | 0.94 | (0.80 – 1.11) | 0.91 | (0.74 – 1.11) | 0.92 | (0.75 – 1.14) | 0.92 | (0.77 – 1.08) |
| 14 | 0.92 | (0.80 – 1.05) | 0.90 | (0.73 – 1.10) | 0.92 | (0.79 – 1.06) | 0.90 | (0.72 – 1.11) |
| 21 | 0.91 | (0.77 – 1.08) | 0.91 | (0.77 – 1.07) | 0.91 | (0.77 – 1.08) | 0.91 | (0.78 – 1.06) |
| 30 | 0.89 | (0.76 – 1.04) | 0.86 | (0.69 – 1.07) | 0.88 | (0.74 – 1.05) | 0.85 | (0.68 1.07) |

^a^ Bland-Altman analysis after logarithmic transformation, then back-transformation.

Abbreviations: BD: Becton Dickinson; CD4: Cluster of Differentiation Type 4; EDTA: Ethylenediaminetetraacetic acid; LoA: Limits of Agreement.
